# Supplementary material for: Sutureless Purely Off-Clamp Robot-Assisted Partial Nephrectomy: Avoiding Renorrhaphy Does Not Jeopardize Surgical and Functional Outcomes
Source: Cancers (Basel). 2023 Jan 23;15(3):698. doi: 10.3390/cancers15030698 (PMC9913582; doi:10.3390/cancers15030698)
Supplement: Supplementary file 1 [file cancers-15-00698-s001.zip › cancers-2106893-supplementary.pdf]

Table S1: baseline characteristics and outcomes of patients intraoperatively converted from SL to RR ocRAPN

	**Intraoperatively converted from SL to RR** n=10
Age, yrs	56 (51-70)
Male gender, n (%)	8 (80%)
ASA ≧ 3, n (%)	1 (10%)
Baseline eGFR, ml/min	88.7 (78.5-96.8)
Tumor Size, cm	4.7 (3-6)
cT2, n(%)	2 (20%)
RENAL score	
≤ 6	0 (0%)
7-9	6 (60%)
≥ 10	4 (40%)
LOS, d	2 (2-3)
Blood Transfusions, n (%)	1 (10%)
Postoperative eGFR, ml/min	71.6 (62.6-79.7)
Trifecta, n (%)	8 (80%)
PSM, n (%)	0 (0%)
CD ≧ 3 complications, n (%)	1 (10%)
sRFD, n (%)	1 (10%)

SL = sutureless, RR = renorrhaphy, PSM = propensity score matched analysis, ASA = American Society of Anesthesiologists score, eGFR = estimated glomerular filtration rate, LOS = length of stay, PSM = positive surgical margins, CD = Clavien-Dindo grade, sRFD = significant renal function deterioration

Table S2: Uni/-multivariable logistic regression analyses to identify predictors of Significant renal function deterioration (sRFD) after ocRAPN.

	Univariable **Logistic regression analysis** to identify predictors of sRFD				Multivariable **Logistic regression analysis** to identify predictors of sRFD			
	OR	95% CI Lower	95% CI Higher	p	OR	95% CI Lower	95% CI Higher	p
Age	**0.98**	**0.96**	**0.99**	**0.03**	**1.07**	**1.04**	**1.11**	**< 0.001**
Male gender	1.12	0.62	2.01	0.71	-	-	-	-
ASA ≧ 3	1.60	0.86	2.95	0.13	-	-	-	-
Pre-eGFR	0.97	0.97	0.98	<0.001	1.66	0.76	3.65	0.78
RENAL score				**< 0.001**				**< 0.001**
≤ 6	**ref**	**-**	**-**		**ref**	**-**	**-**	
7-9	**1.58**	**0.74**	**3.39**	**0.24**	**1.66**	**0.76**	**3.65**	**0.21**
≥ 10	**4.20**	**1.96**	**9.01**	**< 0.001**	**4.96**	**2.23**	**11.06**	**< 0.001**
Renorrhaphy	**2.31**	**1.13**	**4.71**	**0.02**	**2.35**	**1.12**	**4.94**	**0.02**

ASA = American Society of Anesthesiologists score, Pre-eGFR = preoperative estimated glomerular filtration rate
